# OsMADS1 Interacts with OsMADS22 and OsYABBY5 to Regulate Floral Organ and Meristem Identity in Rice

**DOI:** 10.3390/plants15081271

**Published:** 2026-04-21

**Authors:** Hongyan Shen, Xinhao Zhang, Yali Chen, Ruihua Mao, Yiyan Chen, Yuanyi Hu, Xinqi Li

**Affiliations:** 1 School of Tropical Agriculture and Forestry, Hainan University, Haikou 570228, China; 17863808135@163.com (H.S.); 18226011161@139.com (Y.C.); mrh0708@163.com (R.M.); 13466231220@163.com (Y.C.); 2 National Center of Technology Innovation for Saline-Alkali Tolerant Rice in Sanya, Sanya 572024, China; 3 College of Horticulture, Hunan Agricultural University, Changsha 410128, China; 17267298729@163.com; 4 State Key Laboratory of Hybrid Rice, Hunan Hybrid Rice Research Center, Changsha 410125, China

**Keywords:** rice, floral organ identity, floral meristem, *OsMADS1*, protein interaction

## Abstract

The development of rice flowers and panicles critically affects grain yield and quality. *LEAFY HULL STERILE1/OsMADS1*, a grass-specific *SEPALLATA*-like MADS-box transcription factor, is essential for rice floral development and floral meristem activity maintenance. However, the mechanism through which *OsMADS1* interacts with other genes to regulate floral organ identity and meristem determinacy remains unclear. In this study, we first generated *OsMADS1* knockout mutants using CRISPR/Cas9. The mutant florets exhibited obvious morphological defects, which were categorized into five phenotypic classes. Yeast two-hybrid screening identified two OsMADS1-interacting proteins: OsMADS22, an STMADS11-like protein, and OsYABBY5, a YABBY transcription factor. Their physical interactions were validated both in vitro and in vivo, and were important for floral organ specification and meristem maintenance. Transcriptomic analysis revealed that *OsMADS1* regulates numerous genes involved in hormone signaling and panicle/flower development. Furthermore, OsMADS1 acts together with OsMADS22 and OsYABBY5 to modulate the expression of the downstream target *OsMADS55*, thereby controlling rice spikelet development. Together, our results reveal that OsMADS1 executes diverse regulatory functions in floral organ specification and meristem identity by interacting with multiple developmental regulators, providing new insights into the molecular mechanisms of plant flower development.

## 1. Introduction

Angiosperms, or flowering plants, represent the most widespread, diverse, and adaptable group in the plant kingdom, primarily divided into dicots and monocots. The flower, a distinctive reproductive organ of angiosperms, exhibits remarkable morphological diversity and structural complexity, and is critical for survival and seed formation [1]. Since the 1990s, molecular genetic studies on floral development have been conducted in the model dicots Arabidopsis thaliana and Antirrhinum majus. A large number of floral organ development mutants have been identified and analyzed, and the corresponding regulatory mechanisms have been extensively characterized. These studies led to the establishment of the classic ABCDE model of floral development [2,3,4,5,6,7,8]. In most dicots, such as Arabidopsis, flowers typically contain four whorls: four sepals at the outermost, followed by four petals, six stamens, and a central pistil [9,10]. Five classes of genes (A, B, C, D, E) individually or cooperatively control the development of these floral organs, elucidating the molecular basis of floral organ identity. In Arabidopsis, the key genes identified include the A-class genes *APETALA1* (*AP1*) and *APETALA2* (*AP2*), the B-class genes *APETALA3 (AP3*) and *PISTILLATA* (*PI*), the C-class gene *AGAMOUS* (*AG*), the D-class genes *SEEDSTICK* (*STK*), *SHATTERPROOF1* (*SHP1*), and *SHP2*, and the E-class genes *SEPALLATA1/2/3/4* (*SEP1/2/3/4*). Studies show that the ABCDE model of floral development also applies to monocot grasses like rice, maize, and wheat, with the underlying molecular mechanisms being more conserved in these grasses than in Arabidopsis [11].

Rice is a model monocot plant and one of the world’s most important food crops. The development of rice flowers and panicles is closely related to the three yield components, and significantly influences the final grain yield. The rice inflorescence is a panicle, with the spikelet serving as the basic structural unit. Each spikelet consists of one fertile floret, two sterile lemmas (also called empty glumes), and two glumes. The fertile floret is composed of an outer lemma and an inner palea, two small and translucent lodicules, six stamens, and one pistil made up of carpels, stigmas, and ovary. In contrast to many dicots, the first whorl of floral organs in rice comprises the lemma and palea, while the second, third, and fourth whorls are lodicules, stamens, and pistil respectively. Studies have shown that the ABCDE model in rice is similar to that in *Arabidopsis* with respect to regulating floral organ development. Most B-, C-, D-, and E-class genes in rice perform conserved or semi-conserved functions during floral morphogenesis [12,13]. However, some genes, especially class A and E genes, differ substantially in function from their counterparts in *Arabidopsis* [14,15].

Most ABCDE genes encode MADS-box transcription factors, which contain the conserved MIKC domain. The MADS-box motif within this domain is highly conserved [16]. In rice, A-class genes include *OsMADS14*, *OsMADS15*, and *OsMADS18* [17]. *OsMADS14* and *OsMADS15* are key regulators of floral meristem formation and the specification of lemma and palea identity [18]. B-class genes consist of *OsMADS2*, *OsMADS4*, and *OsMADS16* (*SUPERWOMAN1*). *OsMADS2* regulates lodicule development independently of *OsMADS4,* whereas OsMADS16 interacts with OsMADS4 to control stamen formation [19]. C-class genes in rice are represented by *OsMADS3*, *OsMADS58*, and *DL*. Loss of *OsMADS3* leads to stamens to transform into lodicule-like structures, accompanied by ectopic lodicule formation [20]. *OsMADS58* plays a role in carpel development and floral meristem determinacy [21]. *DL*, a member of the YABBY gene family, is indispensable for pistil identity in rice [22]. Two D-class genes, *OsMADS13* and *OsMADS21*, have been identified in rice. *OsMADS13* specifically regulates ovule morphogenesis and floral meristem determinacy. In contrast, *OsMADS21* has lost its essential function in ovule identity and meristem determination during evolution [13,23]. A number of class E genes have been characterized in rice: *OsMADS1/LHS1*, *OsMADS5*, *OsMADS34/PAP2*, *OsMADS7*, and *OsMADS8*. *OsMADS1/LHS1* has important functions in lemma/palea formation, floral meristem determinacy, and floral organ identity [24,25]. Mutations in *OsMADS34/PAP2* cause obvious defects in inflorescence and spikelet morphology, indicating its essential function in their development [26]. *OsMADS7* and *OsMADS8* show functional redundancy during spikelet development; concurrent loss of both genes results in severe floral organ abnormalities and the loss of floral determinacy [27].

*OsMADS1/LHS1*, a rice E-class gene, plays multiple critical roles in rice floral organ development. Mutations in *OsMADS1/LHS1* lead to severe floral organ defects, including elongated and open leaf-like palea and lemma, extra leafy lodicules, reduced stamen number, increased carpel number, and extra florets on the same rachilla. These observations indicate that *OsMADS1/LHS1* is essential for the development of palea and lemma as well as floral meristem determinacy [24,28]. As an E-class protein, OsMADS1 physically interacts with the B-class protein OsMADS16/SPW1, the C-class protein OsMADS58, and the D-class protein OsMADS13 to regulate rice floret development. *OsMADS1* partly contributes to the specification of lodicules and stamens by mediating the *OsMADS16/SPW1* regulatory pathway. Its interaction with OsMADS58 prevents the reversion of the spikelet meristem and is crucial for floral meristem determinacy. *OsMADS1* regulates meristem determinacy partially independent of *OsMADS13* [29]. In addition, OsMADS1 interacts with other floral development-related genes to modulate rice floret development. For instance, its interaction with OsNF-YB9 leads to spikelet developmental defects [30], and its interaction with LONG STERILE LEMMA (G1), a DUF640 family protein, is important for sterile lemma development [31].

Plant development is shaped not only by gene regulatory networks but also by dynamic phytohormone signaling pathways. Recent findings indicate that phytohormones operate as interconnected modules rather than discrete routes, forming complex transcriptional circuits that integrate both developmental cues and environmental signals [32,33]. This hormonal crosstalk is crucial for coordinating floral organ identity and meristem activity—processes also under the control of MADS-box transcription factors. Hence, deciphering how factors like OsMADS1 interface with broader regulatory frameworks, including hormonal pathways, is key to understanding the molecular basis of floral development in rice. Despite extensive studies on OsMADS1 interactions, the mechanisms by which it coordinates with non-MADS-box regulators and hormonal signals remain unclear. We hypothesize that OsMADS1 forms transcriptional complexes with specific regulatory proteins (both MADS-box and non-MADS-box) to control floral organ identity and meristem determinacy via downstream gene modulation, and that these complexes integrate with phytohormone pathways to coordinate floral development.

To test this hypothesis, in this study, we employed CRISPR/Cas9 to generate *OsMADS1* knockout mutants, which exhibited obvious floral organ defects. OsMADS22 and OsYABBY5 were found to be interacting proteins of OsMADS1, and their physical interactions were validated both in vitro and in vivo. Transcriptomic analysis revealed that *OsMADS1* modulates multiple genes involved in hormone signaling and panicle development. We further demonstrated that OsMADS1 functions together with OsMADS22 and OsYABBY5 to regulate the downstream gene *OsMADS55* during rice spikelet development. These results reveal that OsMADS1 collaborates with various developmental regulators to govern floral organ identity and meristem determinacy, which broadens our understanding of the regulatory network of flower development in plants.

## 2. Results

### 2.1. The OsMADS1-Cas9 Mutant Exhibits Defects in Spikelet Organ Specification and Meristem Identity

**Figure 1:**
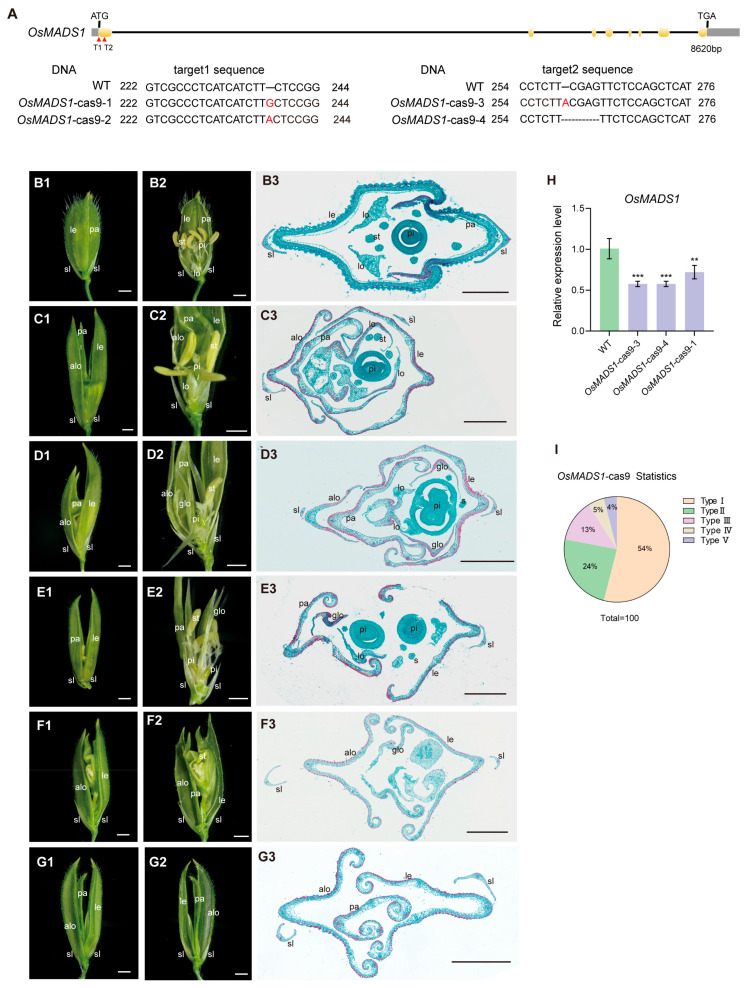
Genotypic and phenotypic analyses of CRISPR/Cas9-derived *osmads1* mutants. (**A**) Target sites in *OsMADS1* using CRISPR/Cas9 genome editing. The positions of the two target sites are indicated in the gene structure. Base deletions and insertions are represented by black dashed lines and red letters, respectively. (**B1**) A complete spikelet of the wild type. (**B2**) Half the lemma and palea from (**B1**) were removed. (**B3**) Cross section of the wild-type spikelet. (**C1**–**G3**) Five distinct mutant phenotypes of *OsMADS1*-cas9. (**C1**–**C3**) Type I floret showing glume-like organs and reduced stamen number. (**C2**) Half of the lemma, palea, and additional lemma/palea-like organs from (**C1**) were removed. (**C3**) Cross-section of the Type I spikelet. (**D1**–**D3**) Type II floret exhibiting elongated lodicules, glume-like organs, and reduced stamen number. (**D2**) Half of the lemma, palea, and additional lemma/palea-like organs from (**D1**) were removed. (**D3**) Cross-section of the Type II spikelet. (**E1**–**E3**) Type III spikelet bearing twin florets. (**E2**) Half of the lemma and palea from (**E1**) were removed. (**E3**) Cross-section of the Type III spikelet. (**F1**–**F3**) Type IV floret with an ectopic rachilla in the central region. (**F2**) Half of the lemma, palea, and additional lemma/palea-like organs from (**F1**) were removed. (**F3**) Cross-section of the Type IV spikelet. (**G1**–**G3**) Type V floret showing complete loss of the inner three whorls of floral organs. (**G2**) Half of the lemma, palea, and additional lemma/palea-like organs from (**G1**) were removed. (**G3**) Cross-section of the Type V spikelet. (**H**) Expression level analysis of *OsMADS1* in wild-type and *OsMADS1*-cas9 mutant panicles. (**I**) Statistical analysis of the five spikelet types in *OsMADS1*-cas9 mutants. Alo: additional lemma/palea-like organ; glo: extra glume-like second whorl organ; le: lemma; lo: lodicule; pa: palea; pi: pistil; sl: sterile lemma; st: stamen. Scale bars = 1 mm (**B1**–**G1**,**B2**–**G2**), 500 µm (**B3**–**G3**). Values are means ± SD (*n* = 3); significance analysis was performed with Students *t*-test (** *p* < 0.01, *** *p* < 0.001). The same wild-type (WT) control image was reused in Figures 4C, 5A and 6A.

To elucidate the biological function of *OsMADS1*, we designed two target sites, T1 (GTCGCCCTCATCATCTTCTCCGG) and T2 (CCTCTTCGAGTTCTCCAGCTCAT), within the first exon of the gene in this study. We generated double-target knockout lines of *OsMADS1* in the japonica rice cultivar Zhonghua 11 (ZH11) using CRISPR/Cas9 editing. After PCR identification and Sanger sequencing, four types of homozygous knockout lines were obtained: insertions of G or A at target site 1, and an A insertion or CGAG deletion at target site 2 (Figure 1A). QRT-PCR analysis confirmed that *OsMADS1* expression was drastically reduced in these knockout lines (Figure 1H). At the heading stage, spikelets of wild-type and *OsMADS1* knockout mutants were observed under a stereomicroscope. No significant differences were observed in rudimentary glumes and sterile lemmas between wild-type and mutants (Figure 1). However, severe defects were found in inner floral organs. Based on phenotypic severity, mutant spikelets were classified into five types. Type I spikelets accounted for 54% (*n* = 100), showing multiple elongated lemma/palea-like structures in the outer whorls, reduced stamens, normal carpels and stigmas, and a pair of extra glume-like organs in the inner whorls (Figure 1(C1–C3),I). Type II spikelets (24%, *n* = 100) exhibited elongated and widened lodicules, increased stigmas, reduced stamens, and additional glume-like organs (Figure 1(D1–D3),I). Type III spikelets (13%, *n* = 100) contained two adjacent florets instead of one apical floret, with reduced stamens, no lodicules, and multiple glume-like organs (Figure 1(E1–E3),I). Type IV spikelets (5%, *n* = 100) developed an elongated rachilla bearing an ectopic spikelet (Figure 1(F1–F3),I). Type V spikelets (4%, *n* = 100) showed lemma/palea-like organs in outer whorls but complete loss of the inner three whorls of floral organs (Figure 1(G1–G3),I).

### 2.2. Ectopic Expression of OsMADS1 Led to Elongated Glumes

**Figure 2:**
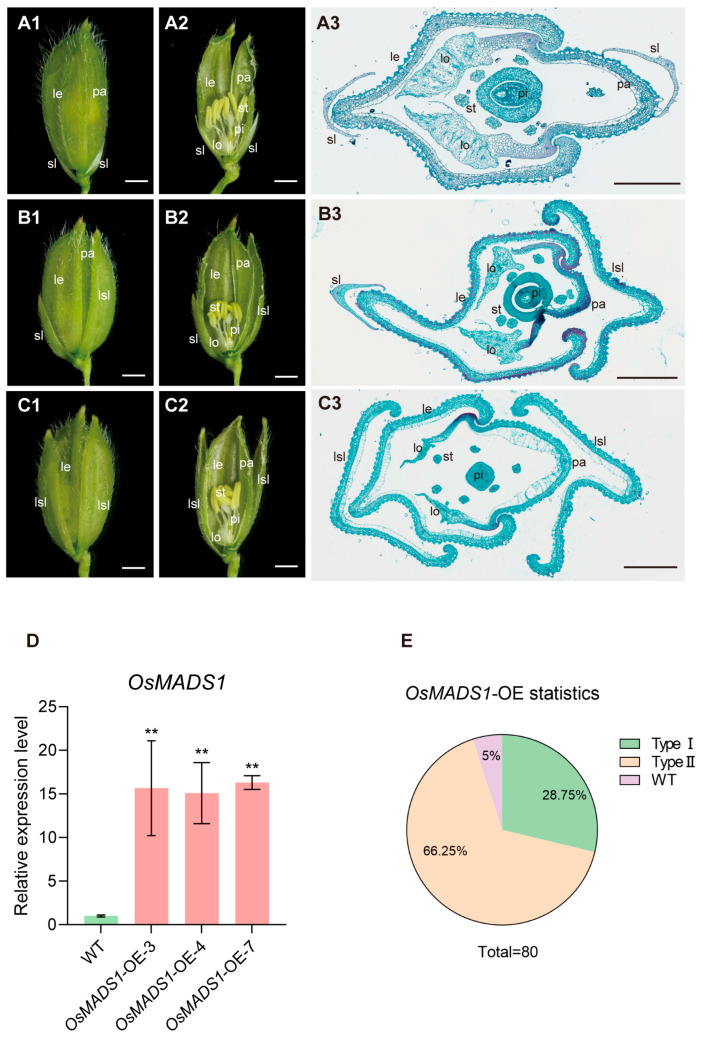
Phenotypes of spikelets in wild-type (WT) and *OsMADS1*-OE plants. (**A1**) A complete spikelet of the wild type. (**A2**) Half the lemma and palea from (**A1**) were removed. (**A3**) Cross section of the wild-type spikelet. (**B1**) A sterile lemma was elongated of *OsMADS1*-OE spikelet. (**B2**) Half the lemma, palea, and elongated sterile lemma from (**B1**) were removed. (**B3**) Transverse section of *OsMADS1*-OE spikelet with one sterile lemma elongated. (**C1**) Two sterile lemmas were elongated of *OsMADS1*-OE spikelet. (**C2**) Half the lemma, palea, and elongated sterile lemma from (**C1**) were removed. (**C3**) Transverse section of *OsMADS1*-OE spikelet with two sterile lemmas elongated. (**D**) Expression level analysis of *OsMADS1* in WT and *OsMADS1*-OE panicles. (**E**) Statistical data of *OsMADS1*-OE spikelet types. le: lemma; lo: lodicule; pa: palea; pi: pistil; sl: sterile lemma; lsl: elongated sterile lemma; st: stamen. Scale bars = 1 mm (**A1**–**C1**,**A2**–**C2**), 500 µm (**A3**–**C3**). Values are means ± SD (*n* = 3); significance analysis was performed with Students *t*-test (** *p* < 0.01).

To better understand the function of *OsMADS1*, we generated *OsMADS1*-overexpressing (*OsMADS1*-OE) transgenic lines in the japonica rice cultivar Zhonghua 11 (ZH11) under the control of the maize ubiquitin promoter. QRT-PCR analysis confirmed that *OsMADS1* transcript levels were significantly upregulated in multiple homozygous *OsMADS1*-OE lines compared to the wild type (Figure 2D). Four independent, high-expressing homozygous lines were selected for further phenotypic analysis. Under a stereomicroscope, the rudimentary glumes and florets of the *OsMADS1*-OE plants appeared normal, whereas sterile lemmas were markedly elongated. Specifically, 28.75% had one elongated and one normal sterile lemma (Figure 2(B1–B3),E), 66.25% of spikelets exhibited a pair of elongated sterile lemmas (Figure 2C1–C3,E), and only 5% retained normal sterile lemmas (Figure 2(A1–A3),E). This result indicates that ectopic expression of *OsMADS1* specifically promotes the elongation of sterile lemmas.

### 2.3. OsMADS1 Interacts with OsMADS22 and OsYABBY5

**Figure 3:**
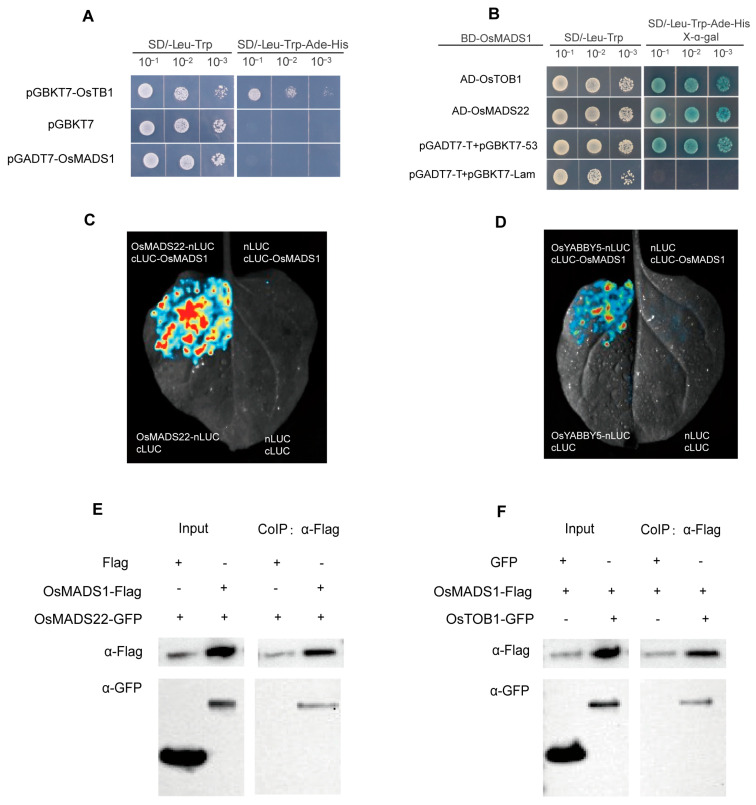
OsMADS1 physically interacts with OsMADS22 and OsYABBY5. (**A**) Yeast auto-activation activity assays of OsMADS1, pGBKT7-OsTB1 and pGBKT7 were used as positive and negative controls, respectively. (**B**) Yeast two-hybrid assay showing that OsMADS1 interacts with OsMADS22 and OsYABBY5. pGBKT7-53 + pGADT7-T and pGBKT7-Lam + pGADT7-T were used as positive and negative controls, respectively. (**C**,**D**) Split firefly luciferase complementation imaging (LCI) assay showing that OsMADS1 interacts with OsMADS22 (**C**) and OsYABBY5 (**D**) in *N. benthamiana* leaves. (**E**,**F**) Co-IP analyses showing that OsMADS1 interacts with OsMADS22 (**E**) and OsYABBY5 (**F**).

To further investigate the transcriptional activity of OsMADS1 protein, we performed a transactivation assay using the yeast two-hybrid system. The full-length coding sequence of *OsMADS1* was cloned into the pGBKT7 vector. The known transcription activator pGBKT7-OsTB1 served as a positive control, and the empty pGBKT7 vector served as a negative control. These constructs were separately transformed into yeast Y2HGold competent cells. Single colonies were picked, resuspended, and spotted onto SD/-Trp/-His/-Ade triple-dropout medium to examine growth. Yeast cells harboring pGBKT7-OsTB1 grew well on both SD/-Trp and SD/-Trp/-His/-Ade media (Figure 3A). In contrast, cells carrying the empty pGBKT7 or pGBKT7-OsMADS1 failed to grow on the SD/-Trp/-His/-Ade medium (Figure 3A). These results demonstrate that OsMADS1 exhibits no transcriptional auto activation activity.

To explore the molecular mechanisms of *OsMADS1* in floral development, we used pGBKT7-OsMADS1 as bait in a yeast two-hybrid screen against a previously constructed cDNA library from rice inflorescences at Sp6-Sp8 stages. Co-transformation of the bait vector with the library into Y2HGold yeast cells yielded normal growth on non-selective SD/-Trp/-Leu medium, while six clones turned blue on selective SD/-Trp/-Leu/-His/-Ade/X-α-gal medium, indicating positive interactions. PCR amplification and sequencing of these clones followed by BLAST (https://blast.ncbi.nlm.nih.gov (accessed on 10 December 2025)) analysis identified their corresponding genes. Two candidates were selected for further study: *LOC_Os02g52340* (*OsMADS22*), an SVP-like MADS-box gene involved in spikelet determinacy [34], and *LOC_Os04g45330* (*OsYABBY5*), a YABBY transcription factor related to *Arabidopsis FILAMENTOUS FLOWER* that plays a critical role in spikelet development [35]. Yeast co-transformation assays, with OsMADS22 and OsYABBY5 cloned into pGADT7, confirmed physical interactions between OsMADS1 and both proteins (Figure 3B). Firefly luciferase complementation imaging (LCI) in *Nicotiana benthamiana* leaves further verified these interactions in planta: strong fluorescence was detected in leaves co-expressing nLUC-OsMADS22/cLUC-OsMADS1 or nLUC-OsYABBY5/cLUC-OsMADS1, but not in control combinations (Figure 3C,D). Co-immunoprecipitation (Co-IP) assays, using transiently co-expressed OsMADS1-Flag with OsMADS22-GFP or OsYABBY5-GFP in tobacco leaves, showed that both OsMADS22-GFP and OsYABBY5-GFP co-precipitated with OsMADS1-Flag, but not with the negative control protein (Figure 3E,F). Collectively, these data confirm that OsMADS1 interacts with OsMADS22 and OsYABBY5 both in vitro and in vivo.

### 2.4. The OsMADS22-Cas9 Mutant Exhibits No Significant Defects and OsYABB5-Cas9 Mutant Exhibits Visible Defects in Spikelet Organ Development

**Figure 4:**
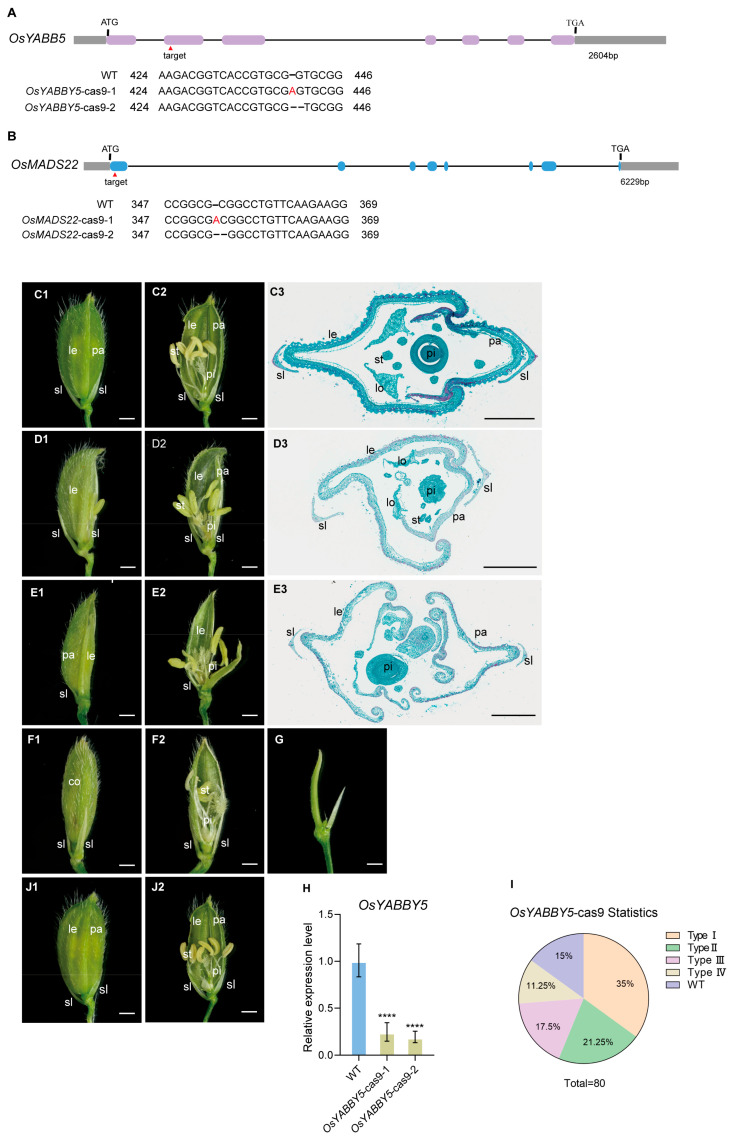
Phenotypic analyses of *OsYABBY5*-cas9 and *OsMADS22*-cas9 mutants. (**A**) CRISPR/Cas9-mediated mutagenesis of *OsYABBY5* and (**B**) *OsMADS22*. The positions of one target sites are indicated in the gene structure. Base deletions and insertions are represented by black dashed lines and red letters, respectively. (**C1**) Spikelets of the wild type. (**C2**) Half of the lemma and palea were removed from C1. (**C3**) Cross section of the wild-type spikelet. (**D1**) A Type I *OsYABBY5*-cas9 spikelet with reduced palea. (**D2**) Half the lemma and palea from (**D1**) were removed. (**D3**) Cross-section of the Type1 spikelet. (**E1**) A Type II *OsYABBY5*-cas9 spikelet lacking obvious lemma and sterile lemma. (**E2**) Half of the lemma from (**E1**) was removed. (**E3**) Cross-section of the Type II spikelet. (**F1**) A Type III *OsYABBY5*-cas9 spikelet forming cone-like organs. (**F2**) Half of the lemma from (**F1**) was removed. (**G**) A Type IV *OsYABBY5*-cas9 spikelet showing complete loss of the inner three whorls of floral organs. (**H**) Expression levels analysis of *OsYABBY5* in wild-type and *OsYABBY5*-cas9 mutant panicles. (**I**) Statistical data of *OsYABBY5*-cas9 mutant spikelet types. (**J1**) Spikelets of *OsMADS22*-cas9 mutant plants. (**J2**) Half of the lemma and palea were removed from (**J1**). le: lemma; lo: lodicule; pa: palea; pi: pistil; sl: sterile lemma; co, cone-shaped organ; st: stamen. Scale bars = 1 mm (**A1**–**D1**,**A2**–**D2**), 500 µm (**A3**–**D3**). Values are means ± SD (*n* = 3); significance analysis was performed with Students *t*-test (**** *p* < 0.0001).

To investigate the roles of the OsMADS1-interacting proteins, we designed sgRNA target sites in the first exons of *OsYABBY5* (AAGACGGTCACCGTGCGGTGCGG) and *OsMADS22* (CCGGCGCGGCCTGTTCAAGAAGG), and constructed *OsYABBY5*-CRISPR/Cas9 and *OsMADS22*-CRISPR/Cas9 knockout vectors. These were transformed into wild-type ZH11 to generate *OsYABBY5*-cas9 and *OsMADS22*-cas9 transgenic plants. PCR and sequencing identified an A insertion or G deletion at the *OsYABBY5* target site and an A insertion or C deletion at the *OsMADS22* target site. (Figure 4A,B). At the heading stage, spikelet morphology was examined in the knockout lines., Four distinct spikelet types appeared in *OsYABBY5*-cas9 mutants. Type I florets showed defective paleae, which were reduced or absent to varying degrees, accounting for 35% (*n* = 80) (Figure 4(D1–D3),I). Type II florets (approximately 21.25%, *n* = 80) lacked obvious lemmas and sterile lemmas, with reduced stamens and 2–3 stigmas per pistil (Figure 4(E1–E3),I). Type III (17.5%, *n* = 80) displayed cone-like organs derived from lemmas, with reduced stamens and normal pistils (Figure 4(F1,F2),I). Type IV (11.25%, *n* = 80) showed complete loss of inner floral organs and abolished floret identity (Figure 4G,I). By contrast, no obvious phenotypic differences were observed between wild-type and *OsMADS22*-cas9 plants (Figure 4(C1–C3,J1,J2)).

### 2.5. Ectopic Expression of OsMADS22 Led to Elongated Glumes and Aberrant Palea

**Figure 5:**
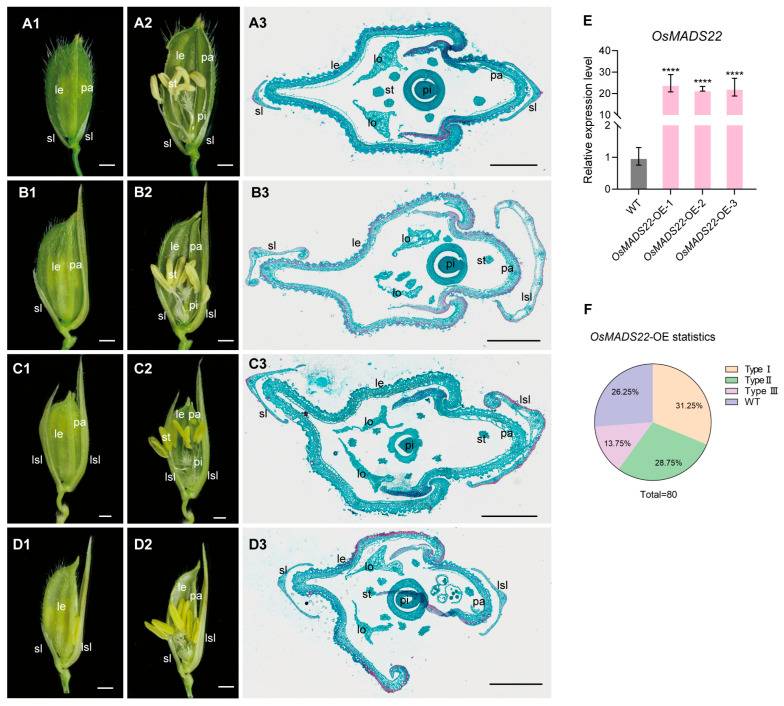
Phenotypes of spikelets in wild-type (WT) and *OsMADS22*-OE plants. (**A1**) A complete Spikelet of the wild type. (**A2**) Half the lemma and palea from the spikelet in (**A1**) were removed. (**A3**) Transverse section of the wild-type spikelet. (**B1**) A sterile lemma was elongated in the *OsMADS22*-OE spikelet. (**B2**) Half the lemma, palea, and elongated sterile lemma from the spikelet in (**B1**) were removed. (**B3**) Transverse section of *OsMADS22*-OE spikelet with one sterile lemma elongated. (**C1**) Two sterile lemmas were elongated in the *OsMADS22*-OE spikelet. (**C2**) Half the lemma, palea, and elongated sterile lemma from the spikelet in (**C1**) were removed. (**C3**) Transverse section of *OsMADS22*-OE spikelet with two sterile lemmas elongated. (**D1**) Reduced palea size and one elongated sterile lemma were observed in the *OsMADS22*-OE spikelet. (**D2**) Half the lemma, palea, and elongated sterile lemma from the spikelet in (**D1**) were removed. (**D3**) Transverse section of *OsMADS22*-OE spikelet with reduced size of the palea (**E**) Analysis of *OsMADS22* expression levels in WT and *OsMADS22*-cas9 panicles. (**F**) Statistical data of *OsMADS22*-OE spikelet types. le: lemma; lo: lodicule; pa: palea; pi: pistil; sl: sterile lemma; lsl: elongated sterile lemma; st: stamen. Scale bars = 1 mm (**A1**–**D1**,**A2**–**D2**), 500 µm (**A3**–**D3**). Values are means ± SD (*n* = 3); significance analysis was performed with Students *t*-test (**** *p* < 0.0001).

To further investigate the function of *OsMADS22*, we amplified its full-length cDNA and placed it under the control of the maize ubiquitin promoter. The Ubi::*OsMADS22* construct was transformed into ZH11 via *Agrobacterium*-mediated transformation. Four independent homozygous *OsMADS22*-OE lines were selected for further analysis, all of which showed significantly higher *OsMADS22* expression than the wild type (Figure 5E). The *OsMADS22*-OE spikelets exhibited defects in floral organ development, which were categorized into three types: Type I florets (31.25%, *n* = 80) exhibited markedly elongated glumes on the palea side, while the lemma side was unaffected (Figure 5(B1–B3),F). Type II florets (28.75%, *n* = 80) showed significantly elongated glumes on both the lemma and palea sides (Figure 5(C1–C3),F). Type III florets (13.75%, *n* = 80) displayed narrow and underdeveloped palea (Figure 5(D1–D3),F). Notably, the floral phenotypes observed in *OsMADS22*-OE plants, particularly the glume elongation, closely resembled those previously described for *OsMADS1*-OE plants. This phenotypic similarity suggests that *OsMADS22* and *OsMADS1* may function in a common pathway to regulate spikelet meristem determinacy in rice.

### 2.6. Ectopic Expression of OsYABB5 Results in Aberrant Spikelet Organ and Meristem Identity

**Figure 6:**
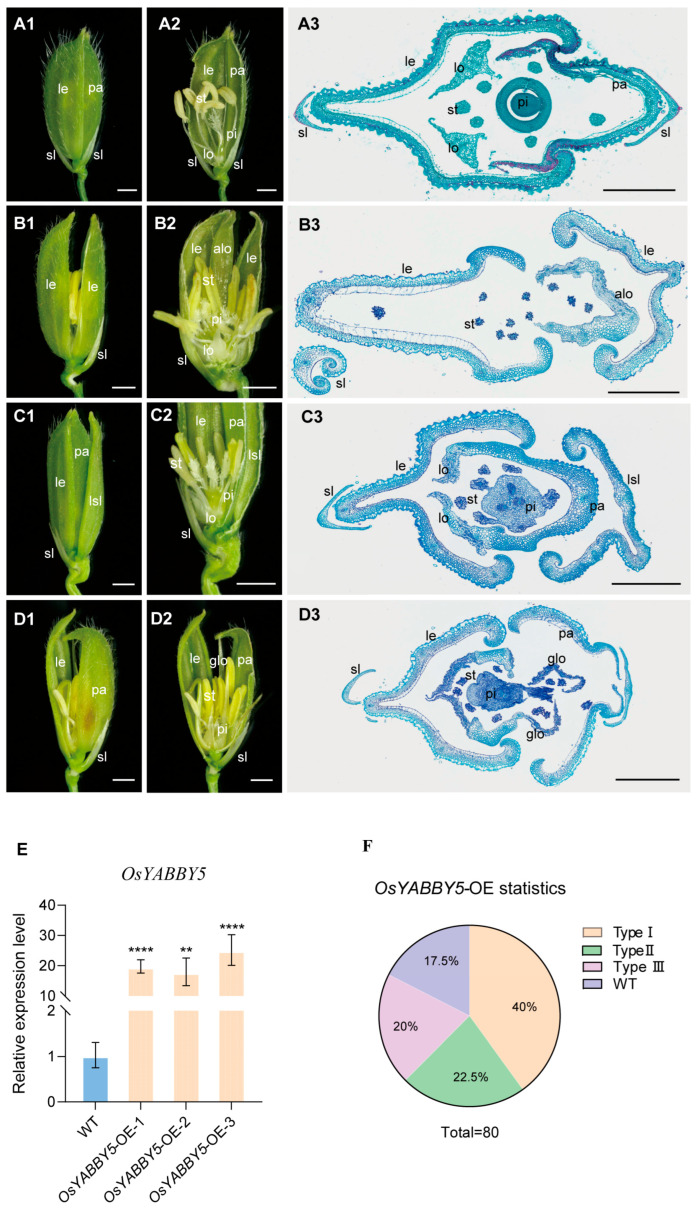
Phenotypes of spikelets in wild-type (WT) and *OsYABBY5*-OE plants. (**A1**) A complete Spikelet of the wild type. (**A2**) Half the lemma and palea from the spikelet in (**A1**) were removed. (**A3**) Transverse section of the wild-type spikelet. (**B1**) A spikelet was unclosed with additional lemma/palea-like organ in the *OsYABBY5*-OE plants. (**B2**) Half the lemmas from the spikelet in (**B1**) was removed. (**B3**) Transverse section of the unclosed spikelet. (**C1**) A spikelet formed an extra lemma/palea-like organ with increased stamens and stigmas.in the *OsYABBY5*-OE plants. (**C2**) Half the lemma, palea, and additional lemma/palea-like organ from the spikelet in (**C1**) were removed. (**C3**) Transverse section of the spikelet with extra lemma/palea-like organ. (**D1**) The spikelet shows indistinct palea, glume-like organs, and an increased stamen number in the *OsYABBY5*-OE spikelet. (**D2**) Half the palea from the spikelet in (**D1**) was removed. (**D3**) Transverse section of the spikelet with indistinct palea and extra lemma/palea-like organ. (**E**) Analysis of *OsYABBY5* expression levels in WT and *OsYABBY5*-OE panicles. (**F**) Statistical data of *OsYABBY5*-OE spikelet types. Alo: additional lemma/palea-like organ; glo: extra glume-like second whorl organ; le: lemma; lo: lodicule; pa: palea; pi: pistil; sl: sterile lemma; st: stamen. lsl: elongated sterile lemma; Scale bars = 1 mm (**A1**–**D1**,**A2**–**D2**), 500 µm (**A3**–**D3**). Values are means ± SD (*n* = 3); significance analysis was performed with Students *t*-test (** *p* < 0.01,**** *p* < 0.0001).

To further investigate the function of OsYABBY5, we generated *OsYABBY5*-overexpressing transgenic rice plants in the Zhonghua 11 background under the control of the maize ubiquitin promoter. QRT-PCR analysis of young panicles confirmed significantly higher *OsYABBY5* expression in the *OsYABBY5*-OE lines than in wild-type ZH11 (Figure 6E). Four homozygous transgenic lines were chosen for further phenotypic analysis. *OsYABBY5*-OE plants exhibited three types of defective florets. Type I florets (40%, *n* = 80) remained unclosed due to an increased number of floral organs, including extra stamens, pistils, and lodicules (Figure 6(B1–B3),F). Type II florets (22.5%, *n* = 80) had one normal glume and one glume that developed into a lemma-like structure; histological sections confirmed that this modified glume acquired lemma identity. These florets also showed increased stamen and stigma number (Figure 6(C1–C3),F). Type III florets (20%, *n* = 80) showed homeotic transformation of palea into a lemma-like structure, with the palea losing its distinct identity and glume-like organs forming inside the floret, accompanied by an increased number of stamens and lodicules (Figure 6(D1–D3),F). These results indicate that ectopic expression of *OsYABBY5* disrupts normal floral organ identity, number, and closure.

### 2.7. Transcriptome Analysis of OsMADS1-OE Plants by RNA-Seq

**Figure 7:**
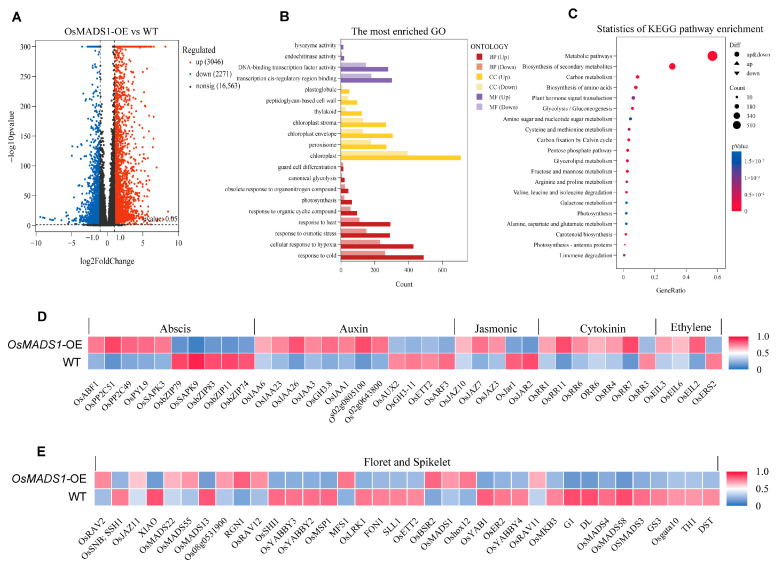
Transcriptome analysis of wild type (WT) and *OsMADS1*-OE plants. (**A**) Volcanic plot of the differentially expressed genes. (**B**) Gene Ontology (GO) enrichment analysis. (**C**) KEGG pathway enrichment analysis of differentially expressed genes. (**D**) Heatmap of DEGs involved in plant hormone signal pathways in wild-type (WT) and *OsMADS1*-OE plants. (**E**) Heatmap of DEGs Involved in floret development in wild-type (WT) and *OsMADS1*-OE plants.

To further explore the potential mechanism by which *OsMADS1* regulates floral meristem determinacy and floral organ identity, we collected young inflorescences (≤2 cm) from *OsMADS1*-overexpressing (*OsMADS1*-OE) and wild-type plants at the booting stage, with three biological replicates for each genotype. Total RNA was extracted and subjected to transcriptome sequencing after quality validation. Compared with the wild type, 5317 differentially expressed genes (DEGs) were detected in *OsMADS1*-OE lines, including 3046 upregulated and 2271 downregulated genes (Figure 7A). KEGG pathway enrichment analysis revealed that the most significantly enriched pathways were “Metabolic pathways” and “Biosynthesis of secondary metabolites” (Figure 7C). Several DEGs were also enriched in plant hormone signal transduction pathways, mainly involving abscisic acid, auxin, jasmonic acid, cytokinin, and ethylene, with the majority of DEGs participating in the auxin signaling pathway (Figure 7D). Furthermore, multiple key regulators of rice spikelet and flower development were identified among the DEGs. These included *OsMADS1* (regulating lemma/palea development); *OsMADS3*, *OsMADS58*, and *DL* (controlling stamen and pistil identity); *OsMADS13* (specifically regulating ovule development); *OsMADS4* (modulating lodicule development); *OsMADS22* and *OsMADS55* (affecting meristem indeterminacy and floral development); *OsYABBY3*, *OsYABBY4*, and *OsYAB1* (regulating reproductive meristem maintenance and fate as well as spikelet development); and *FON1* (controlling floral organ number) (Figure 7E).

### 2.8. The Protein Interactions of OsMADS1 with OsMADS22 and OsYABBY5 Regulates Spikelet Development by Attenuating the OsMADS1-Mediated OsMADS55 Transcriptional Activation

**Figure 8:**
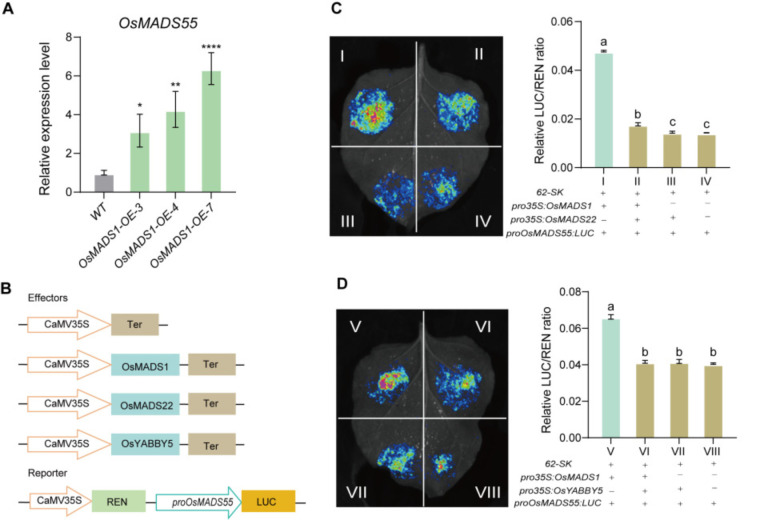
OsMADS1 interacts with OsMADS22 and OsYABBY5 to attenuate transcriptional activation of OsMADS55. (**A**) Expression levels of *OsMADS55* in developing young panicles of wild-type and OsMADS1-OE plants, determined by RT-qPCR (*n* = 3). (**B**) Schematic illustration of LUC/REN assay used for transcriptional activity analysis. The 35S-driven REN gene serves as an internal control for normalization. The effector of empty vector was used as the control. (**C**,**D**) Transient transcriptional activity assays in *N. benthamiana* leaves showing that OsMADS22 (**C**) and OsYABBY5 (**D**) attenuate the OsMADS1-mediated activation of the *OsMADS55* promoter. Values are means ± SD (*n* = 3); significance analysis was performed with Students *t*-test (* *p* < 0.05, ** *p* < 0.01, **** *p* < 0.0001). Different letters indicate significant differences (*p* < 0.05, one-way ANOVA, Tukey’s test). I–VIII represent different combinations, as shown in the figure.

*OsMADS1* is a MADS-box transcription factor. Previous studies have shown that it can directly regulate the expression of downstream target genes such as *OsMADS17*, *OsMADS55*, *OsKANADI4*, *OsPIN1*, and *OsHB4*. Consistent with this, our transcriptome analysis of young inflorescences from *OsMADS1*-OE lines and wild-type plants revealed significantly upregulated *OsMADS55* expression in *OsMADS1*-OE lines (Figure 7E). To further investigate how the interaction between *OsMADS1* and *OsMADS22* or *OsYABBY5* regulates *OsMADS55* expression and influences spikelet development, we first performed RT-qPCR analysis. The results confirmed that *OsMADS55* transcript levels were significantly upregulated in the *OsMADS1*-OE lines (Figure 8A). We then conducted a transient transcriptional activation assay, which showed that the LUC activity driven by the *OsMADS55* promoter was induced by OsMADS1. However, this activation was significantly attenuated when OsMADS1 was co-expressed with either OsMADS22 or OsYABBY5 (Figure 8C,D). Collectively, these findings demonstrate that OsMADS1 interacts with OsMADS22 and OsYABBY5 to attenuate its own transcriptional activation activity. This regulatory module fine-tunes the expression of the downstream target gene *OsMADS55*, thereby influencing rice spikelet development.

## 3. Discussion

Previous studies on *OsMADS1* mutant alleles, such as *afo*, *nsr*, and *lhs1*, have established its unique and conserved role in specifying lemma and palea identity in grass florets [18,28,36]. Defective florets in *OsMADS1*-cas9 knockout mutants partially were consistent with previously reported allelic mutants. A defining feature is the elongation and abnormal leaf-like differentiation of the lemma and palea, while the empty and rudimentary glumes remain unaffected (Figure 1(D1,F1)), underscoring the whorl-specific function of *OsMADS1.* Our observations align with prior reports that *OsMADS1* overexpression induces sterile lemma elongation [37], a phenotype also observed in the *g1/M15* long sterile lemma mutant [38]. Upregulated *OsMADS1* expression in *g1/M15* sterile lemmas further supports the notion that G1 and OsMADS1 interact to co-regulate sterile lemma development. A key feature of the rice palea is the marginal region of the palea (MRP), whose smooth epidermal cells allow the lemma and palea to lock together, enclosing the inner floral organs. As seen with other loss-of-function alleles, the MRP in some of our *OsMADS1*-cas9 mutant florets lost its distinct identity and acquired lemma/palea body (BOP)-like characteristics (Figure 1). This defect likely contributes to the improper closure of the floret observed in the mutants.

Our *OsMADS1* mutants exhibited floral meristem determinacy defects, consistent with the established roles of C- and D-class MADS-box genes in rice. In 13% of mutant spikelets, the single terminal floret was replaced by two closely associated florets, often with two carpels (Figure 1(E1–E3)). Another 4% of florets showed failure of floral meristem termination, resulting in continuous production of leaf-like lemma/palea organs (Figure 1(G1–G3)). This loss of determinacy points to a role for *OsMADS1* in repressing meristem activity, a core function of C-class genes. Rice C-class genes *OsMADS3* and *OsMADS58* have partially redundant but specialized roles, with expression domains overlapping *OsMADS1* at the early meristem apex. While *osmads58* single mutants are normal, the *osmads3 osmads58* double mutant displays indeterminate florets with repeated lodicule-like organs, confirming their combined role in meristem termination. Extra carpels in our *OsMADS1* mutants (Figure 1(D1–D3,E1–E3)) link its function to D-class activity: the D-class gene *OsMADS13* is essential for ovule development, and its mutation induces ectopic carpels. Given their in vitro interaction, OsMADS1 and OsMADS13 likely form an in vivo complex to repress meristem activity, specify ovule identity, and prevent ectopic carpel formation. Most floral MADS-box proteins act in higher-order complexes. We propose that OsMADS1 functions within a higher-order MADS-box complex containing C-class (*OsMADS3/OsMADS58*) and D-class (*OsMADS13*) factors to achieve coordinated termination of the floral meristem and specify organ identity. Disruption of this complex in *OsMADS1* mutants leads to the observed indeterminacy.

Various floret defects in *OsMADS1*-cas9 mutants provide further evidence that *OsMADS1* is critical for lemma/palea formation and floral meristem determinacy. As an E-class protein, OsMADS1 interacts with A-class proteins OsMADS15 and the AGL6 protein OsMADS6. The OsMADS1-OsMADS15 interaction is vital for meristem determinacy, as *osmads1 osmads15* double mutants switch from reproductive to vegetative growth, converting florets into new plants [18]. OsMADS1 also interacts with OsMADS6 to regulate inner whorl floral organ development [39]. Furthermore, OsMADS1 genetically and physically interacts with B-, C-, and D-class proteins to enable coordinated regulation of meristem identity and floral organ specification [29]. Through yeast two-hybrid, luciferase complementation, and co-immunoprecipitation assays, we demonstrate OsMADS1 physically interacts with OsMADS22 and OsYABBY5 both in vitro and in vivo (Figure 3). Previous studies have shown that both OsMADS1 and OsYABBY5 are highly expressed in young inflorescences [35], and *OsYABBY5* expression is downregulated in *OsMADS1*-knockdown spikelets [24]. In agreement with this, *OsMADS1*-cas9 and *OsYABBY5*-cas9 knockout mutants generated in this study share similar phenotypes, including lemma/palea defects, reduced stamens, pistil abnormalities, and severe loss of inner floral organs and floret identity. These shared critical roles in floral organ development further support a functional interaction between *OsMADS1* and *OsYABBY5*. Notably, *OsMADS1*-cas9 knockout mutants have normal spikelets, but *OsMADS22* overexpression induces sterile lemma elongation, mimicking *OsMADS1* overexpression (Figure 5). This suggests OsMADS22 cooperates with OsMADS1 in sterile lemma development and exhibits functional redundancy with other floral regulators.

Our transcriptome data show that *OsMADS1* overexpression alters the expression of more than 5300 genes, with clear enrichment in pathways linked to hormone signalling, particularly auxin, and secondary metabolism (Figure 7). These changes coincide with misregulation of known floral identity genes such as *OsMADS3*, *OsMADS58*, *DL*, and *FON1*. Based on these results, we propose a working model: *OsMADS1* helps maintain floral meristem determinacy and correct organ specification partly by modulating auxin-related signals and by directly or indirectly controlling a set of spikelet- and flower-specific transcription factors. However, a clear limitation of this study is the lack of direct molecular evidence for *OsMADS1* target genes. RNA-seq alone cannot distinguish direct transcriptional targets from downstream secondary effects. Without chromatin immunoprecipitation (ChIP)-based validation or equivalent methods such as DAP-seq or yeast one-hybrid, we cannot confirm which of the observed expression changes are caused by direct binding of OsMADS1 to promoter regions. Therefore, our current analysis provides a global transcriptional landscape and generates testable hypotheses rather than defining a definitive regulatory hierarchy. Future work combining ChIP-seq with targeted gene perturbation will be essential to identify genuine direct targets and to fully understand how *OsMADS1* orchestrates rice flower development.

Morphological and histological analyses of *OsMADS1*-Cas9 mutants, together with previous functional studies, reinforce the essential role of *OsMADS1* in floral organ development and the maintenance of floral meristem activity. Transcriptome analysis of young inflorescences from *OsMADS1*-overexpressing lines and wild-type plants identified multiple differentially expressed genes involved in rice flower/spikelet development. Among them was *OsMADS55*, a known regulator of meristem indeterminacy. It has been reported that *OsMADS55* is a direct downstream target of *OsMADS1* [24]. *OsMADS1* and *OsMADS55* overexpression lines share similar spikelet phenotypes, such as elongated sterile lemmas and shortened panicle branches [40]. Notably, *OsMADS55* expression was significantly upregulated in *OsMADS1*-OE lines (Figure 8A), indicating that *OsMADS1* positively regulates *OsMADS55* expression. This upregulation is consistent with the similar regulatory mechanisms in spikelet development. Here, we show that OsMADS1 interacts with OsMADS22 and OsYABBY5 separately to form transcriptional regulatory complexes. These interactions attenuate the transcriptional activation activity of OsMADS1, thereby fine-tuning the expression of its downstream target *OsMADS55* during spikelet development. How OsMADS22 and OsYABBY5 individually attenuate the transcriptional activation activity of OsMADS1 is still unclear, but several possibilities could be considered. OsMADS22, being a MADS-box protein, might compete with OsMADS1 for CArG-box sites in the promoter of target genes such as *OsMADS55*. If OsMADS22 occupies those sites, OsMADS1 binding and activation would be attenuated. Alternatively, OsMADS22 and OsYABBY5 can separately assemble with OsMADS1 into higher-order transcriptional complexes. In either case, these complexes do not recruit RNA polymerase II or co-activators efficiently, so transcriptional output goes down. Another possibility is that OsMADS22 or OsYABBY5 acts as a scaffold, bringing co-repressors like TPL/TPR family proteins or histone deacetylases to OsMADS1 target sites. These co-repressors then attenuate transcription by altering chromatin structure or interfering with activator function.

In summary, we generated various *OsMADS1* knockout lines in rice using CRISPR/Cas9 gene editing technology, all knockout lines showed varying floral defects. We further demonstrated that OsMADS1 physically interacts with both OsMADS22 and OsYABBY5 in vitro and in vivo. These interactions fine-tune the transcriptional activation activity of OsMADS1, thereby regulating the expression of its downstream target *OsMADS55* to control spikelet organ development and meristem determinacy. These findings provide valuable insights for further investigating the molecular mechanisms by which *OsMADS1* regulates floral development. Biologically, the physical interaction between OsMADS1and OsYABBY5 adds a new layer to how MADS-box and YABBY proteins coordinate floral meristem determinacy and organ identity. Agriculturally, because spikelet architecture directly impacts grain yield, adjusting OsMADS1 activity or its protein interactions could improve panicle structure and floret fertility. Taken together, our results not only offer new insights into how *OsMADS1* regulates floral development but also point toward new direction for breeding higher-yielding rice.

## 4. Materials and Methods

### 4.1. Plant Materials and Growth Conditions

The rice variety Zhonghua 11 was used as the genetic background. Three CRISPR-Cas9 knockout mutants, namely *OsMADS1*-cas9, *OsMADS22*-cas9, and *OsYABBY5*-cas9, were generated by Wuhan Bioway Biotechnology Co., Ltd. After transformation, target sites were sequenced to obtain homozygous lines. Both wild-type and mutant plants were grown at two field locations: the Changsha station of the Hunan Hybrid Rice Research Center and the Sanya station in Hainan Province. Seeds were surface-sterilized and germinated at room temperature (about 25 °C) on wet filter paper. Sprouted seeds were transferred to pre-prepared seedling beds. Around four weeks later, seedlings at the four-leaf stage were transplanted to paddy fields at a spacing of 20 cm between rows and 16 cm within rows. Routine field practices included regular irrigation, balanced fertilization (150-75-75 kg/ha N-P-K), and manual weed and pest control. During the reproductive stage, young inflorescences and other tissues were collected for analysis.

### 4.2. Morphological and Histological Observation

During the reproductive stage, young spikelets from both wild-type and mutant plants were collected and dissected under a stereomicroscope. Each spikelet was examined for its external morphology and internal floral organ structure. The number of spikelets per panicle, the types of spikelets (e.g., normal, abnormal), and the condition of florets (lemma/palea, lodicules, stamens, pistils) were recorded and photographed. For each mutant line, spikelet phenotypes were counted from at least 20 individual plants. For histological observation, freshly harvested young spikelets were immediately submerged in a pre-chilled FAA solution (50% ethanol, 0.9 M glacial acetic acid, 3.7% formaldehyde) and kept at 4 °C for no less than 16 h. Afterwards, the fixed tissues were dehydrated through a series of increasing ethanol concentrations (70%, 85%, 95%, and 100%), cleared in xylene, and infiltrated with paraffin [41]. The embedded tissues were cut into 8-μm-thick sections using a rotary microtome (Leica RM2245, Germany). Sections were then dewaxed with xylene, rehydrated through a descending ethanol series, and stained with 1% safranine (Amresco, Framingham, MA, USA) followed by 1% Fast Green (Amresco). Finally, stained sections were examined and imaged under a light microscope.

### 4.3. Total RNA Isolation and RT-qPCR Analysis

Young spikelet tissues from wild-type and mutant plants were ground in liquid nitrogen. Total RNA was extracted using the FastPure Universal Plant Total RNA Isolation Kit (Vazyme Biotech, Nanjing, China). For each sample, 1 μg of total RNA was reverse-transcribed with the HiScript II Q Select RT SuperMix (Vazyme) at 50 °C for 15 min. Quantitative PCR was performed on an ABI 7500 system (Thermo Fisher Scientific, Waltham, MA, USA) using AceQ qPCR SYBR Green Master Mix (Vazyme). The cycling protocol was: 95 °C for 5 min, then 40 cycles of 95 °C for 10 s and 60 °C for 30 s. The rice *ACTIN* gene (*OsRac1*, LOC_Os01g12900) was used as the internal control. Three biological replicates were analyzed for each sample, and each biological replicate had three technical replicates. Relative expression levels were calculated using the 2^−ΔΔCT^ method. Primer sequences are listed in Table S1.

### 4.4. Transgene Vector Construction and Rice Transformation

To generate *OsMADS1*-cas9, *OsMADS22*-cas9, and *OsYABBY5*-cas9 knockout mutants, we designed single guide RNA (sgRNA) sequences targeting the exon of each gene using the CRISPR-GE online tool (http://skl.scau.edu.cn/). Each sgRNA was cloned into the binary vector pC1300-Ubi::Cas9. For overexpression constructs, the full-length coding sequences of *OsMADS1*, *OsMADS22* and *OsYABBY5* were amplified from rice cDNA using gene-specific primers (listed in Table S1). The PCR products were then inserted into the pCUbi1390 vector [42] downstream of the rice ubiquitin promoter, yielding pUbi::*OsMADS1*, pUbi::*OsMADS22*, and pUbi::*OsYABBY5* fusion constructs. All cloning steps were verified by Sanger sequencing. The final binary vectors were introduced into *Agrobacterium tumefaciens* strain EHA105. Transformation of rice calli (cultivar Zhonghua 11) was carried out by Wuhan Boyuan Biotechnology Co., Ltd. (Wuhan, Hubei, China) following a standard *Agrobacterium*-mediated protocol. Transgenic plants were regenerated on selective media containing hygromycin (50 mg/L) and subsequently transferred to soil. Homozygous knockout lines were identified by PCR and sequencing of the target sites. The relevant primers developed for vector construction are shown in Table S1.

### 4.5. Yeast Two-Hybrid System Assays

The full-length coding sequence of *OsMADS1* was amplified and inserted into the bait vector pGBKT7. This construct (pGBKT7-*OsMADS1*) was then transformed into the yeast strain *Saccharomyces cerevisiae* Y2HGold using a standard lithium acetate method. To check for auto-activation, transformants were first grown on SD/−Trp medium at 30 °C for 3 days, then replica-plated onto SD/−Trp/−His/−Ade medium and incubated for another 3 days. For interaction assays, the coding sequences of *OsMADS1*, *OsMADS22*, and *OsYABBY5* were each cloned into pGBKT7 (bait) or pGADT7 (prey) as appropriate. The bait and prey plasmids were co-transformed into Y2HGold cells and plated onto SD/−Trp/−Leu double dropout (DDO) medium to select for successful co-transformants. After 3 days of growth at 30 °C, colonies were transferred onto high-stringency QDO medium (SD/−Trp/−Leu/−His/−Ade) and cultured for an additional 3 days. Protein interactions were scored based on colony growth on QDO plates. The combination pGADT7-T + pGBKT7-53 was used as a positive control, and pGADT7-T + pGBKT7-Lam as a negative control. All primer sequences used for vector construction are listed in Table S1.

### 4.6. Co-Immunoprecipitation Assay

The coding sequences of *OsMADS22* and *OsYABBY5* were separately inserted into pCAMBIA1300-GFP to produce *OsMADS22*-GFP and *OsYABBY5*-GFP. The *OsMADS1* coding sequence was cloned into pCAMBIA1300-Flag to generate *OsMADS1*-Flag. Each construct was transformed into *Agrobacterium tumefaciens* strain GV3101. For transient expression, bacterial suspensions carrying different construct combinations were mixed to a final OD_600_ of 0.6 and co-infiltrated into *Nicotiana benthamiana* leaves. After 48 h, infiltrated leaf discs were harvested and ground in liquid nitrogen. Total protein was extracted using a lysis buffer (50 mM Tris-HCl pH 7.5, 150 mM NaCl, 1 mM EDTA, 1% Triton X-100, and protease inhibitors). The lysate was cleared by centrifugation at 12,000× *g* for 10 min at 4 °C. The supernatant was incubated with anti-GFP nanobody-conjugated agarose beads for 2 h at 4 °C with gentle rotation. Beads were washed four times with the same lysis buffer. Bound proteins were eluted by boiling in SDS loading buffer. Both input samples and immunoprecipitates were resolved by SDS-PAGE and detected by immunoblotting using anti-GFP and anti-FLAG antibodies. Primers used are listed in Table S1.

### 4.7. Split Firefly Luciferase Complementation Imaging (LCI) Assays

The full-length coding sequence of *OsMADS1* was inserted into pCAMBIA1300-cLUC to generate cLUC-*OsMADS1*. The coding sequences of *OsMADS22* and *OsYABBY5* (without stop codons) were separately cloned into pCAMBIA1300-nLUC to produce nLUC-*OsMADS22* and nLUC-*OsYABBY5*. Each construct was transformed into *Agrobacterium tumefacien* strain GV3101. Bacterial suspensions were adjusted to OD_600_ = 0.5. For each interaction test, cLUC-*OsMADS1* was mixed with nLUC-*OsMADS22* or nLUC-*OsYABBY5* at a 1:1 ratio, and then combined with a p19-expressing strain (OD_600_ = 0.2). The mixture was infiltrated into *Nicotiana benthamiana* leaves. After 48 h, the abaxial side of the infiltrated leaves was sprayed with 1 mM D-luciferin. Following a 5 min dark incubation, luminescence was detected using a cooled CCD camera. Primers are listed in Table S1.

## Data Availability

The original contributions presented in this study are included in the article/Supplementary Materials. Further inquiries can be directed to the corresponding authors.

## Supplementary Materials

The following supporting information can be downloaded at: https://www.mdpi.com/article/10.3390/plants15081271/s1, Table S1: Primers used in the present study; Table S2: DEGs between the wild-type and *OsMADS1*-OE plants; Table S3: GO enrichment analysis of differentially expressed genes; Table S4: KEGG pathway enrichment analysis of differentially expressed genes.
